# Is It Still Relevant to Discover New ACE Inhibitors from Natural Products? YES, but Only with Comprehensive Approaches to Address the Patients’ Real Problems: Chronic Dry Cough and Angioedema

**DOI:** 10.3390/molecules28114532

**Published:** 2023-06-02

**Authors:** Sivananthan Manoharan

**Affiliations:** Molecular Pathology Unit, Cancer Research Centre, Institute for Medical Research, National Institutes of Health, Ministry of Health Malaysia, Setia Alam, Shah Alam 40170, Malaysia; sivananthan@moh.gov.my; Tel.: +60-3-3362-7498

**Keywords:** ACE inhibitors, bradykinin, C-domain, N-domain, natural products, side effects

## Abstract

Despite many publications related to the identification of new angiotensin-I-converting enzyme (ACE) inhibitors, especially peptides from natural products, the actual reason/s for why new ACE inhibitors need to be discovered are yet to be fully understood. New ACE inhibitors are pivotal to address serious side effects caused by commercially available ACE inhibitors in hypertensive patients. Despite the effectiveness of commercial ACE inhibitors, due to these side effects, doctors often prescribe angiotensin receptor blockers (ARBs). Recent evidence has shown the benefits of ACE inhibitors over ARBs in hypertensive patients and hypertensive–diabetes mellitus patients. In order to address these side effects, the somatic ACE’s enzyme structures need to be revisited. The peptides isolated from the natural products need to be verified for their stability against ACE and several important gastrointestinal enzymes. The stable peptides sequence with the presence of favourable ACE inhibitory-related amino-acids, such as tryptophan (W), at the C-terminal need to be subjected to molecular docking and dynamics analyses for selecting ACE inhibitory peptide/s with C-domain-specific inhibition instead of both C- and N-domains’ inhibition. This strategy will help to reduce the accumulation of bradykinin, the driving factor behind the formation of the side effects.

## 1. Introduction

The angiotensin-I-converting enzyme (ACE) inhibitor is one of several classes of anti-hypertensive medicine available to reduce the elevated systolic blood pressure (SBP) in high blood pressure patients. It is used to suppress ACE and reduces high blood pressure. ACE drugs have long been recognized as the main treatment in treating high blood pressure and are expected to continue as a standard therapy for essential hypertension [1]. It has been reported that the frequency of cough requiring the withdrawal of ACE inhibitors was described to be 9.3%. The incidence of cough necessitating cessation of ACE inhibitor treatment was identified to be significantly higher among African and Chinese ethnicities [2]. A study that investigated the cessation rates of lisinopril because of cough identified that 34% of Chinese Americans suffered from a chronic cough that was severe enough to permit withdrawal of the ACE inhibitor. This percentage is significantly higher than that of 13%, which was identified in the overall American population [2,3]. A Singaporean retrospective study further confirmed the above-mentioned study, where 30.1% of Singaporean Chinese patients discontinued ACE inhibitors. Furthermore, the cessation of ACE inhibitors was higher in females than males [2]. This proposes that Asians might have a different response to certain medicines. The outcomes of investigations carried out in non-Asian participants might not always be relevant to Asian populations. Researchers must take into consideration the number of Asians in upcoming clinical trials before generalizing the outcomes for any Asian population [2]. In the Singaporean study, 21.2% patients who were originally initiated on ACE inhibitors were finally changed to angiotensin receptor blockers (ARBs). ARBs can be effective, but are not equal to ACE inhibitors for heart failure with decreased left ventricular ejection fraction (LVEF) and therefore are not considered as a first-line treatment [4]. The ACE inhibitor is maintained as the gold standard for inhibition of the renin angiotensin aldosterone system (RAAS) [4], but in a recently published review article based on many meta-analyses, the effects of angiotensin receptor blockers (ARBs) and ACE inhibitors on several important parameters, such as cardiovascular mortality, are comparable. However, when compared to ARBs, the patients prescribed with ACE inhibitors significantly increased in discontinuation of the drug mainly due to the adverse events, whereas ARBs have a better safety profile [5]. Compared with patients prescribed with ARBs, patients who were prescribed ACE inhibitors were about 30% more likely to have a persistent dry cough [6]. This safety profile made ARBs more advantageous compared to ACE inhibitors in treating patients with hypertension. A report published in the Cochrane Library stated that although ARBs are somewhat better tolerated compared to ACE inhibitors, the availability of higher quality information and statistics backing up the utilization of ACE inhibitors, which can prevent mortality, strokes, and heart disease, are needed to be considered before selecting ARBs over ACE inhibitors [7]. 

Many patients suffering with high blood pressure also have diabetes. Approximately 73.6% of patients with diabetes at the age of 18 or above have high blood pressure. ARBs are a common choice for the treatment of high blood pressure, as well as for the prevention of renal problems in sufferers with diabetes, and are the preferred medication in patients who experience cough with the use of ACE inhibitors [8]. Notwithstanding the benefit of ARBs’ safety profiles, a major meta-analysis available in JAMA Internal Medicine exposed that ACE inhibitors decrease all-cause mortality, cardiovascular mortality, and major cardiovascular events in patients with diabetes mellitus, but ARBs had no advantages in these outcomes. Therefore, ACE inhibitors must be considered as a first-line treatment to control or curb excess mortality and morbidity in these people [9]. Another meta-analysis suggests that therapy with an ACE inhibitor exhibited a significant cardiovascular protection aimed at all-cause mortality, cardiovascular death, and key cardiovascular events, while ARBs had no advantages in these results, excluding myocardial infarction. In reflection of high mortality and morbidity, ACE inhibitors were superior to ARBs in patients with high blood pressure and type 2 diabetes mellitus [10]. In another meta-analysis published in PLoS One, it was shown that ACE inhibitors give rise to a more potent improvement of the homeostasis model assessment of insulin resistance (HOMA-IR) in comparison with ARBs among the long-term intervention and high blood pressure patients with diabetes mellitus subgroup [11]. Although ARBs have a good safety profile, ACE inhibitors have additional benefits when it comes to the management of diabetic patients with high blood pressure. Substituting ACE inhibitors for ARBs in diabetic patients experiencing ACE inhibitor-related side effects, such as dry cough and angioedema, is recommended [8]; however, the researchers in this field, especially those specializing in ACE inhibition in hypertension, should focus more sharply on the real need of the patients so that patients can benefit from ACE inhibition and experience no, fewer, or tolerable side effects. The currently available ACE inhibitors at hospitals are non-domain specific inhibitors. This means ACE inhibitors such as lisinopril inhibit both C- and N-domains of ACE and cause the side effects.

## 2. The ACE, Type of ACE, and Its Characteristics

**Figure 1:**
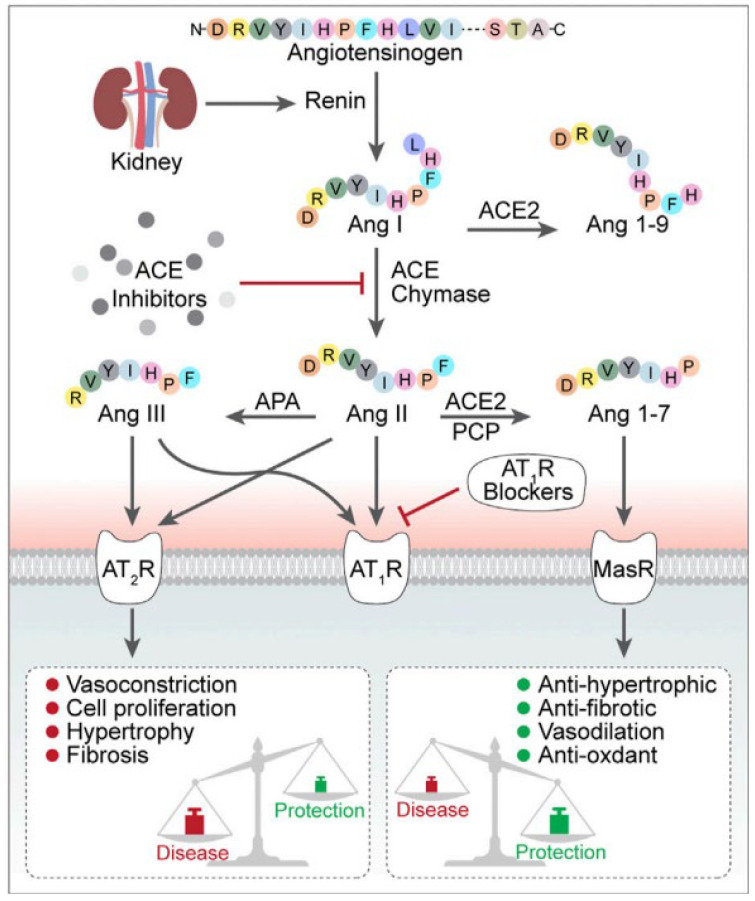
The conversion of angiotensin I to angiotensin II by ACE. Formation of angiotensin II leads to vasoconstriction, cell proliferation, hypertrophy, and fibrosis. (Figure was reproduced from an article with Creative Commons Attribution License (CC BY) [13]).

The ACE is an enzyme present in the renin–angiotensin–system (RAS) and kallikrein–kinin system (KKS), which are involved in regulating human blood pressure. The ACE converts angiotensin I to angiotensin II in RAS. The formation of angiotensin II constricts the blood vessels and increases the blood pressure in humans (Figure 1). On the other hand, ACE converts active bradykinin to inactive bradykinin in KKS. Peptides derived/isolated from natural products have been shown to inhibit ACE. Inhibition of ACE by these peptides leads to blocking the conversion of angiotensin I to angiotensin II and preserves the functionality of bradykinin. Because of ACE inhibition, the vasoconstriction is reversed, and thus reduces the blood pressure [12]. One of the ACE inhibitors’ hallmarks is that inhibitors such as captopril and lisinopril reduce peripheral vascular resistance without changing baroreceptor activity or triggering an increase in the heart rate. At the same time, the ACE inhibitors prevent the tonic effect of angiotensin-II on the sympathetic nervous system [1]. 

There are 3 types of ACE present in humans, known as somatic ACE (sACE), testicular/germinal ACE (tACE), and plasma ACE (pACE). The pACE is a subset of sACE and it forms through cleavage of sACE. The 170 kilo Dalton (kDa) sACE is present in endothelium, epithelial, and neuronal cells. On the other hand, the 100 kDa tACE is mainly found in testis, where it is involved in male reproduction [14]. The sACE and tACE in humans comprises 1306 and 665 amino acids (aa) residues, respectively. The sACE owns two catalytic domains, known as N- and C-domains, while tACE is only composed of one catalytic domain. The N- and C-domains are zinc-metallopeptidase accompanied by active motif HEMGH, where both histidine (H) amino acids coordinate the zinc ion (Zn^2+^) [15]. A large difference between the C- and N-domains is related to their thermal stability. The N-domain was revealed to have a melting temperature (Tm) of 70 °C, which is 15 °C higher than that of the C-domain, which has a Tm of 55 °C, suggesting that the N-domain is more thermostable [16]. The lungs have the maximum quantity of ACE and represent 0.1% of total protein [15]. According to Maluf-Meiken et al. [17], there are 2 isomers that belong to the N-domain of ACE with 65 kDa and 90 kDa molecular weight. It was shown earlier that the N-domain soluble forms of ACE were identified in body fluids mainly in human ileal and human and rats’ urine. The 65 kDa isomer can be found in the urine of both normal and high BP humans and rats, while the 90 kDa isomer is exclusively found in the urine of human and rats with high BP. Based on these findings, the 90 kDa N-domain ACE was proposed as a genetic marker for high BP. The absence of the 90 kDa ACE is evidently linked with various vital aspects in the maintenance of lower levels of BP, which rise with aging. Unlike the population with the 90 kDa isomer, the human population without the 90 kDa isomer can remain at a low proinflammatory status and with little tendency to rise in BP levels. There is not much difference in the N- and C-domain of sACE. In spite of that, N- and C-domains are varied in their chloride dependency, substrate, and inhibitor specificity. It has been identified that the C-domain is largely involved in the regulation of BP. In vitro studies have revealed that the affinity for the substrate angiotensin I is identical for N- and C-domains. However, the catalytic effectiveness for converting angiotensin I to angiotensin II by C-domain is three times greater than that of the N-domain. In vitro studies have shown that N- and C-domains hydrolyse bradykinin by comparable catalytic efficiency. Additionally, it has been shown that blocking the C-domain in vivo stopped the cleavage of angiotensin I. Thus, the C-domain contributes a key function in BP regulation. The blocking of the C-domain is perhaps significant and adequate to treat certain cardiorenal illnesses since this domain is identified as the main site for the formation of angiotensin II [18,19]. In addition, according to van Esch et al. [20], selective inhibition of the C-domain is enough to avert angiotensin I-caused vasoconstriction (narrowing of the blood vessels). In contrast, another study confirmed that selective inhibition of any of the N-domain or the C-domain completely stops conversion of systemically given angiotensin I in mice. It was concurrently shown that when utilising soluble ACE, complete inhibition of angiotensin I cleavage was recorded only during inhibition of both the N- and C-domains [21]. On the other hand, although tACE resembles the C-domain of sACE, it has 36 unique amino acids at the N-terminal [22]. Furthermore, based on the ExPASy Prosite, the TAHHEMGHIQ for the N-domain sACE and the residues VAHHEMGHIQ for the C-domain sACE and tACE with an active motif HEMGH were recognized as the active regions on the proteins [19]. The difference in one amino acid at the N-terminal of both amino acid sequences probably leads to the difference in both substrate and inhibitor specificity.

## 3. ACE Inhibitors, Development of Dry Cough and Angioedema, and Possible Alternative Treatments to Address ACE Inhibitor-Induced Cough

The ACE inhibitor is one of several classes of anti-hypertensive medicine available to reduce elevated systolic blood pressure (SBP) in high blood pressure patients. It is used to suppress ACE and reduces high blood pressure. ACE drugs have long been recognized as the main treatment in treating high BP and are expected to continue as a standard therapy for essential hypertension [1]. ACE blockers are not only used to treated elevated BP, but are also well-known drugs given to patients with heart failure. ACE inhibitors have also been exposed to have a role in keeping the organs from being impaired by illnesses. For instance, when the ACE inhibitor is given alone or in combination, benazepril yields a renal protective effect. In addition, when the increased blood pressure is reduced to a normal reading, benazepril will avert cardiovascular illness [14]. Other than the above-mentioned ACE inhibitor, captopril, lisinopril, and enalaprilat are a few of the well-known ACE inhibitors. Captopril varies from both lisinopril and enalaprilat. This is because its sulphydryl group coordinates the zinc instead of a carboxyl group. Furthermore, its tiny size correspondingly results in fewer direct interactions with ACE. Due to the smaller size, captopril functions at only two positions, in addition to the zinc coordinating group. In term of potencies towards the C-domain of ACE, captopril placed in third position after lisinopril and enalaprilat [1]. According to the Cleveland Clinic, as of August 2021, there are 10 Food and Drug Administration (FDA)-approved ACE inhibitors, known as benazepril, captopril, enalapril, fosinopril, lisinopril, moexipril, perindopril, quinapril, ramipril, and trandolapril. All ACE inhibitors are taken through mouth, but enalapril can be given through an intravenous route as well [23]. In spite of the efficacy of ACE drugs, these drugs have unwelcome side effects, such as coughing, taste disturbance, kidney disease, and angioneurotic oedema [14]. Out of these, the cough is a common problem in patients taking ACE inhibitors. This cough is naturally dry and can be accompanied by a tickling or scratching sensation in the throat. The occurrence of cough related with ACE inhibitors has been described to be in a range of 3.9% and 35%. ACE inhibitor-produced cough might arise within the same day after ingestion of the first dose or may be occur weeks or months later [24]. Interestingly, the degree of the cough is different between the ACE inhibitors. For example, perindopril has a lower than projected frequency of cough when compared to other ACE inhibitors. The real mechanism behind the incidence of cough in patients taking ACE inhibitors is not clearly known, but there are a few theories that have been hypothesized for cough development. The most broadly recognized theory is related to bradykinin and substance P, which are two substrates for ACE, and the complete inhibition of ACE with ACE inhibitors leads to accumulation of these substrates in the upper and lower respiratory tracts. Accumulation of bradykinin encourages sensitization of airway sensory nerves by quickly adjusting the stretch receptors and C-fiber receptors that release neurokinin A and substance P. This makes the airway smooth muscle tighten/narrow, causing bronchoconstriction and cough [25]. The similar accumulation of bradykinin and substance P, which is a peptide that produces vasodilation and fluid release into tissues, are hypothesised for angioedema [26]. Nevertheless, the answer for why cough does not occur in all patients receiving ACE inhibitors still requires further investigations. The additional hypothesized mechanisms consist of bronchial hyperreactivity (BHR), a history of asthma, chronic heart failure (CHF), augmented sensitivity of bradykinin dependent airway sensory nerve fibers, bradykinin receptor gene polymorphism amplified cough reflex sensitivity, aminopeptidase P (APP) enzyme insufficiency to break down bradykinin (similarly hypothesised for angioedema), and mechanisms that comprise of ACE insertion/deletion polymorphism [24,25,26]. 

It has been shown that iron supplementation inhibited cough in patients taking ACE inhibitors. The use of ACE inhibitors can cause the generation of nitric oxide (NO). It was hypothesised that supplementing iron, which is a NO synthase inhibitor, could reduce the incidence of cough in patients taking ACE inhibitors. An iron supplement of 256-mg as a tablet or placebo was given to patients for a therapy period of 4 weeks. Supplementation of iron showed a significant reduction in the scores of coughs, where the mean daily scores were 3.07 ± 0.70 and 1.69 ± 1.10 (*p* < 0.01) during the last week of observation and treatment period, respectively, but not with placebo (2.57 ± 0.80 and 2.35 ± 1.22). Three patients in the iron group presented nearly complete cough elimination [27]. In separate results published by Lancet, the investigators investigated whether the prostanoid thromboxane was involved in the incidence of ACE inhibitors-induced cough. In a double-blind placebo study, while continuing enalapril treatment, picotamide was given to nine subjects suffering from essential hypertension who had a cough after taking enalapril 20 mg once a day. Picotamide is a drug that acts as a platelet antiaggregant, where the drug performs its action through both the inhibition of thromboxane synthase and thromboxane-receptor antagonism. Treatment with picotamide 600 mg twice a day caused the disappearance of the cough in eight subjects within 72 h. A thromboxane antagonist is potent in causing ACE inhibitors-induced cough. A disproportion between thromboxane and prostacyclin might be a possible marker in identifying patients who are susceptible to ACE inhibitor-induced cough [28]. In another interesting discovery, two patients who had dry cough after being treated with lisinopril were switched to another ACE inhibitor known as fosinopril. The patients had no cough related to the ACE inhibitor after the lisinopril was changed to fosinopril [29]. In addition, a 68-year-old non-smoker woman with no respiratory-related diseases experienced continued chronic dry cough within a month of starting the ACE inhibitor quinapril to address her essential hypertension. One month after shifting to fosinopril treatment, the patient testified a full recovery from the cough and remains cough-free [30]. In a prospective, multicenter, randomized, 8-week double-blind therapy, cough occurrence and severity with ACE inhibitors fosinopril and enalapril were evaluated in 179 non-smokers with mild to moderate high blood pressure who had previously experienced an ACE inhibitor-induced cough. Fosinopril and enalapril produced comparable blood pressure control, but with significant improvement in the cough profile, which demonstrates favour for fosinopril. A subgroup analysis showed that all-cough recurrence was 33.5 ± 6.3 vs. 56.6 ± 5.3 (*p* = 0.006) for fosinopril and enalapril, respectively. In conclusion, patients who had an ACE inhibitor-induced cough might experience less frequent cough with fosinopril [31]. In a separate study based on a multicenter, 7-week, prospective, open-label pilot study, which included non-smokers with mild to moderate high blood pressure being treated with ACE inhibitors other than fosinopril and having an active and non-productive cough, replacement of the current ACE inhibitor to fosinopril caused a significant reduction (*p* < 0.001) in the frequency of the cough [32]. 

Based on these points addressing alternative treatments to reduce cough that is caused by ACE inhibitors other than fosinopril, even within the class of ACE inhibitors there are different ACE drugs that lead to different amounts of cough production. Based on these arguments, another field of research has been developed wherein researchers can focus on testing fosinopril in patients suffering from ACE-induced cough. Although some research has been conductive respective to this argument, more studies are needed to confirm the previous findings. It is common in the field of science that the scientists always hear or read the phrase ‘more research is needed to confirm the previous findings or current conclusion.’ This means more quality research is needed. The outcomes from the open-label study, observational study, retrospective study, or studies with less, small, or not up to the minimum requirement of the sample size cannot always impact the current practice. The outcomes from a good design of the studies, such as phase 1 to 3 with clear objective/s, optimal sample sizes, and comparators, together with other requirements, such as randomization, blinding strategies, presence of a placebo, and a minimum risk of bias can impact the practice. Usually, with good outcomes from a well-designed phase 3 study with an optimal sample size often impact the current practice. 

## 4. Peptides’ Stability against Gastrointestinal Enzymes and Its ACE Domain-Specific Inhibition

**Figure 2:**
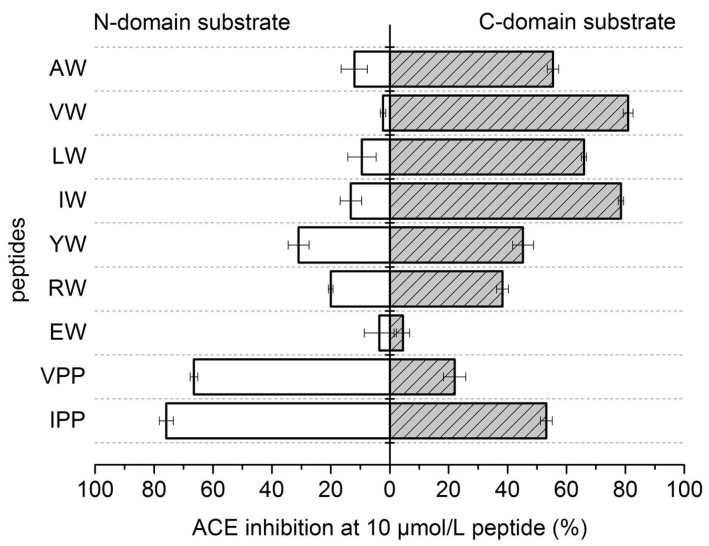
Dipeptide VW as an ACE C-domain specific inhibitor. Note: Peptides with ‘W’ amino acid at the C-terminal mostly act as C-domain inhibitors. (Reproduced with permission from Elsevier. License number: 5483960119076. License date: 7 February 2023 [18].

**Figure 3:**
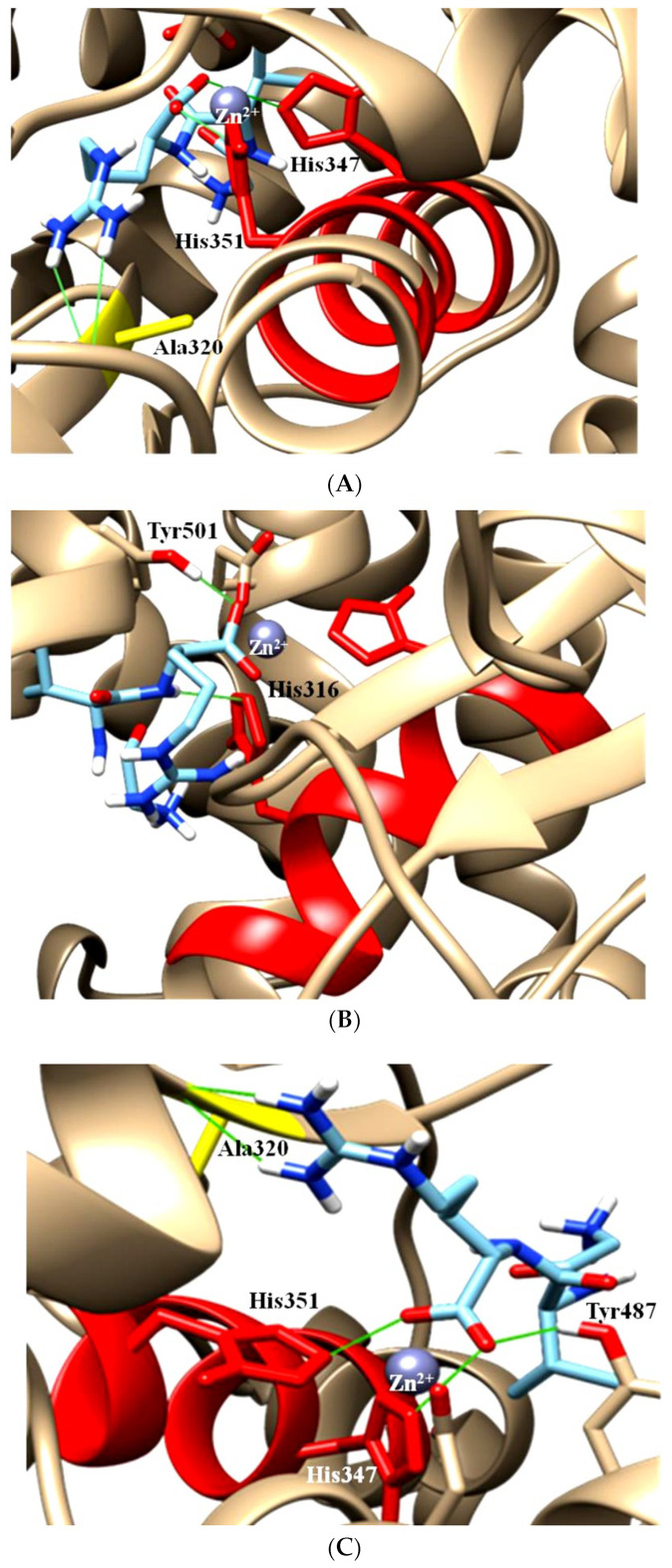
(**A**) Tripeptide GVR blocked (as shown by the green line) both arms of catalytic sites (His347 & His351) of the C-domain. (**B**) Tripeptide GVR blocked only one arm (His316) of the catalytic site of the N-domain, whereas another arm remained open. (**C**) The same peptide blocked both arms in testicular ACE. The docking works were narrowed down using the grid box 50 × 50 × 50 to focus only on this area where the motif HEMGH is present. The motive was to show how tripeptide GVR interacts at the catalytic site. Note: These are remodelled structures where the missing amino acids were added, and the structures were validated through Ramachandran plots. In the non-remodelled C-domain ACE structure, His347 and His351 were known as His383 and His387 (the arms). (The figures were reproduced from an article with Creative Commons Attribution License 4.0 International License [14]).

When an ACE inhibitor inhibits a somatic ACE without domain specificity, accumulation of bradykinin is expected and when the accumulation reaches a certain level, it will be translated into chronic dry cough and angioedema [19]. This is the exact area in which researchers should focus. Based on the above-mentioned reasons in Section 1, ACE inhibitors still have relevance and potency in treating high blood pressure. This opens another interesting opportunity for researchers to discover new ACE inhibitors with C-domain specific inhibition rather than both. By doing this, accumulation of bradykinin can be avoided since the N-domain is not inhibited, thus reducing the side effect/s in patients experiencing chronic dry cough and angioedema. Recently published articles have shown dipeptide VW and tripeptide GVR act more towards or mimic C-domain-specific inhibition of ACE (Figure 2 and Figure 3). Although molecular docking studies have shown tripeptide GVR as a C-domain inhibitor due to blocking of the catalytic arms of the C-domain rather than the N-domain, unlike the previous version, the recently updated BIOPEP database has shown the tripeptide GVR was not stable in Pepsin pH > 2 where this peptide sequence is digested to G-VR. Dipeptide VR is an ACE inhibitor isolated from Atlantic salmon [13]. The in vivo experiment [33,34] using tripeptide GVR was conducted in daily strictly fasted spontaneously hypertensive rats (SHRs) where the GVR was stable in Pepsin pH 1.3 (especially in the morning after fasting overnight), but this approach may lead to incompliance in patients because some patients may mistakenly take the compound after having a meal. This will cause the peptide to be digested to G-VR and possibly G-V-R if more enzymes are released into the gastrointestinal tract due to the presence of food. Furthermore, if the compound needs to be taken twice or thrice daily, it is most likely that the tripeptide GVR will no longer exert ACE inhibition. On the other hand, dipeptide VW is attractive for the further development because this sequence was stable against many enzymes available in the BIOPEP database; however, it needs to be validated through in vitro digestion of the peptide.

The two most potent C-domain and N-domain specific inhibitors are known as RXPA380 and RXP470, and are new phosphinic peptide inhibitors. RXPA380 had shown 3 nmol/L inhibition of the C-domain and 10,000 nmol/L inhibition for the N-domain. On the other hand, RX407 had 7 nmol/L inhibition for the N-domain and 7500 nmol/L for the C-domain of ACE. Both compounds mildly inhibited neprilysin (NEP) at 25,000 and 6000 nmol/L, respectively. In the in vivo study, the authors mentioned that after adjusting the pH to 7, the RXPA380 (0.9, 3, 10, and 30 mg/kg) and RXP407 (10 mg/kg) were given through an intravenous route [21]. To the best of the author’s knowledge and research, although highly potent, these compounds are not included in human trials. The research was conducted in the ClinicalTrials.gov website. The author speculates that, probably due to the requirement of the intravenous injection setting, these compounds are not suitable for daily intake by patients at home to control essential hypertension. It is presumably also difficult to synthesise these compounds and offer them at an affordable price. This is because, very recently, a hybrid structure known as RXPA380-P was generated and the authors claimed the methods used to generate this hybrid structure were more convenient and operationally simple than the solid-phase protocol to synthesize RXPA380. Although the hybrid structure RXPA380-P had a high IC_50_ value of 250 μM for the C-domain and did not effectively inhibit the C- and N-domains, the authors have shown that there are simple and convenient methods to generate this compound [35]. Based on the structure of RXPA380 and VW, it is known that peptide W is important for C-domain inhibition.

**Figure 4:**
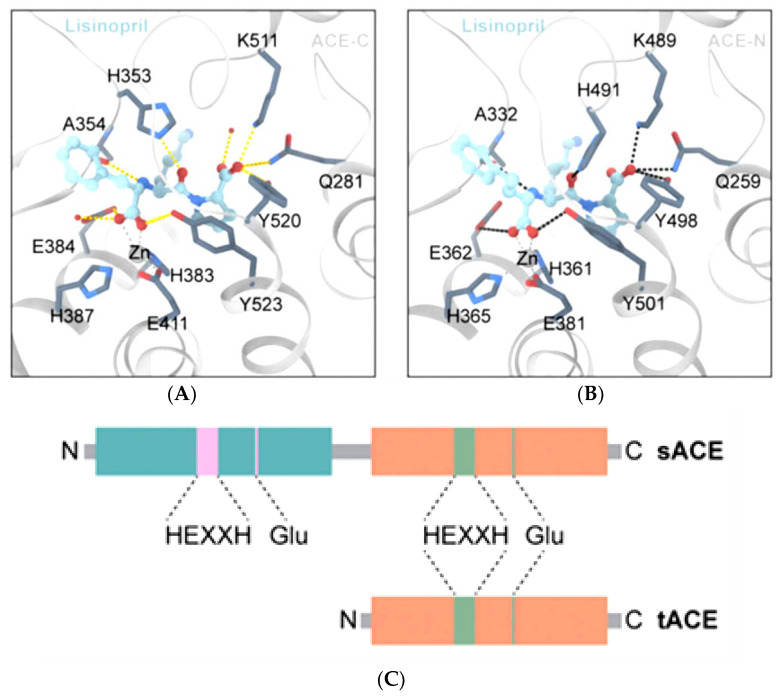
The non-domain specific binding of lisinopril, the gold standard ACE inhibitor, at the C-domain (**A**) and the N-domain (**B**) of ACE. (**C**) The HExxH is the main site of the active site. In the C-domain, this is known as His383-E-x-x-His387, where the His383 and His387 are the catalytic sites of ACE. (The figures were reproduced from an article with Creative Commons Attribution License (CC BY) [13]).

In some published articles, authors used only testicular ACE to carry out molecular docking analysis [36,37]. Although testicular ACE resembles the C-domain of somatic ACE, the absence of the N-domain, which can only be found in somatic ACE, made the analysis inconclusive for determining the ability of the peptide to selectively inhibit the C-domain. Although the C-domain has more of a role in converting angiotensin-I to angiotensin-II, complete inhibition of both the C- and N-domains by a natural peptide will only increase the accumulation of bradykinin. For example, for lisinopril, despite the structural similarity between the C-domain and N-domain of ACE, there are several noteworthy differences among the active sites. E162 in the S1 subunit of the C-domain is substituted by D140 in the N-domain of ACE, but D140 is unable to establish contact with lisinopril. The D377 in the S1 subunit of the C-domain is substituted by Q355 in the N-domain of ACE, but Q355 is far from lisinopril and could not directly contact the lisinopril. These changes might describe why lisinopril has a higher C-domain selectivity [13]. In fact, the C-domain inhibition of lisinopril was 2.4 nM, while for the N-domain, it was 44 nM (IC_50_). Lisinopril has 18 times more selectivity towards the C-domain and is most likely much stronger than angiotensin-I. In Anthony’s study [1], the authors mentioned the IC_50_ of Ang_1-7_, but not angiotensin-I towards the C- and N-domains. Assume the C- and N-domains’ IC_50_ for Ang_1-7_ belong to the C- and N-domain of angiotensin-I (for discussion purposes only; Ang_1-7_ and angiotensin-I are slightly different in terms of the number of amino acids present in the peptide sequences. Refer to Figure 1). In this case, the IC_50_ of angiotensin-I for the C- and N-domains are 130 nM and 3400 nM, respectively, which means angiotensin-I is strongly bound to the C-domain of ACE instead of the N-domain. Although lisinopril is 18 times more C-domain selective, due to its IC_50_ for the C- and N-domain, which is 2.4 nM and 44 nM, respectively, lisinopril can competitively displace angiotensin-I, which is much weaker (in term of IC_50_) than lisinopril and occupies both the C- and N-domains of ACE. This can be found visually in Figure 4A, where lisinopril has a higher C-domain selectivity; lisinopril also interacted with the Zn^2+^ ion in the N-domain ACE (Figure 4B) and blocked the catalytic and active sites. This leads to non-specific domain inhibition of lisinopril. This is another example for why both domains are needed when conducting research related to domain specificity. If dipeptide VW (IC_50_: 1.4–2.5 μM) [38], the potential C-domain ACE inhibitor challenged to compete with angiotensin-I for the domains’ active sites, is able to bind to the N-domain, but not the C-domain, it is because angiotensin-I is weakly bound to the N-domain with IC_50_ of 3.4 μM, but not the C-domain, where the IC_50_ was 130 nM or 0.13 μM. Since scientists have shown that VW prefers the C-domain over the N-domain due to the presence of the amino acid ‘W’ at the C-terminal of the dipeptide, in the case of the active sites of the N-domain not preferring to interact with the dipeptide VW despite VW having good IC_50_ to bind to the N-domain, the interaction of the VW-N domain probably will not occur. It is worth mentioning that although C- and N-domains are dissimilar, the motif HExxH is the same in all three structures (Figure 4C). This means the surrounding of the motif plays an important role in attracting a favourable substrate. 

**Figure 5:**
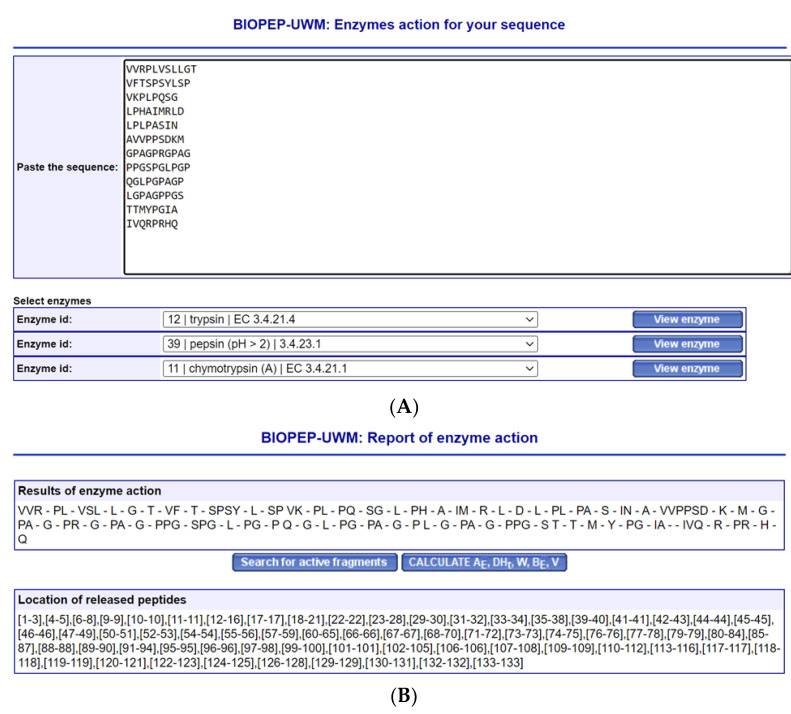
In silico peptide stability against gastrointestinal enzymes. (**A**) The 12 sequences of peptides used for the studies by Abdelhedi et al. [37]. (**B**) The results of the enzymes’ action. All peptides were digested. The results were generated from BIOPEP software [39].

For peptide stability, all 12 peptide sequences listed by Abdelhedi et al. were verified for stability against trypsin, chymotrypsin (A), and pepsin pH > 2 in the BIOPEP database. Based on Figure 5A,B, all peptides appeared to be unstable. Monitoring for the stability of the peptide sequences against gastrointestinal tract enzymes is a vital process if the peptides are meant to be given through the oral route. In fact, if the unstable peptides against gastrointestinal enzymes are used for molecular docking analysis, the output from the in-silico and real-world studies, such as in rodent preclinical experiments or clinical human studies, may be significantly different. The production of fragmented sequences in rodents or humans may no longer exhibit ACE inhibitory activity or domain-specific ACE inhibitory activity. 

## 5. Function of Neprilysin and Chymase

**Figure 6:**
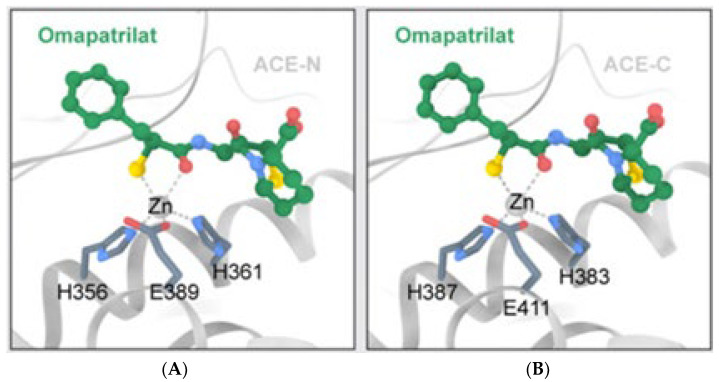
Omapatrilat is a non-domain specific inhibitor. This is because in the N-domain (**A**), the ligand interacted with H356 and H361, while in the C-domain (**B**), the same ligand interacted with H383 and H387. The ligand (omapatrilat) blocked both catalytic sites. This explains why angioedema was observed in the patients. (The figures were reproduced from an article with Creative Commons Attribution License (CC BY) [13]).

**Figure 7:**
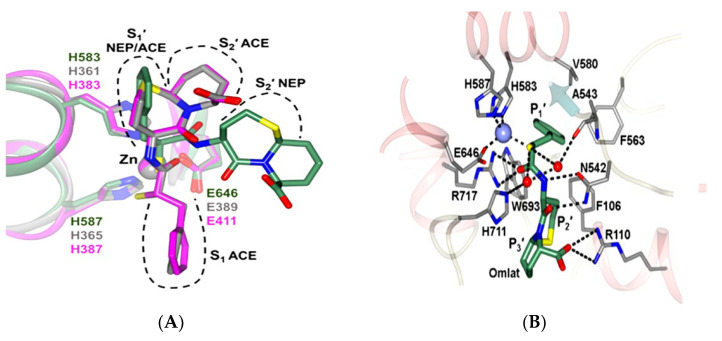
Omapatrilat’s effect in ACE and neprilysin. (**A**) Three structures were overlapped: the C-domain ACE (magenta), the N-domain ACE (gray), and neprilysin (green) are shown. It can be seen that omapatrilat interacted with the main active sites of both enzymes. Indeed, omapatrilat is a dual inhibitor of ACE and neprilysin. (**B**) The binding of omapatrilat to the neprilysin alone. Omapatrilat interacted with H583 and H587. (The figures were reproduced from an article with Creative Commons Attribution License (CC BY) [41]).

Interestingly, while ACE is believed to be the main site of bradykinin degradation, some other enzymes that also include NEP, which is available in high amounts in the kidneys and lungs, similarly lead to bradykinin degradation. NEP also hydrolyses both angiotensin I and angiotensin II. It was proposed that a combination of NEP and ACE inhibitors would be more effective rather than inhibiting one pathway. Subsequently, omapatrilat was developed to inhibit both ACE and NEP, however, the drug failed the clinical trial due to the formation of a side effect of angioedema. Omapatrilat inhibited both domains of sACE and NEP, thus leading to accumulation of bradykinin and, subsequently, leading to angioedema (Figure 6 and Figure 7). This led to the exploration of different approaches that would not affect the bradykinin degradation. One of the approaches is a combination of a C-domain specific ACE inhibitor and a NEP inhibitor [40]. Future studies should not stop at identifying novel ACE inhibitors, but should also demonstrate their stability if they are peptide-based inhibitors and the ability to specifically inhibit the C-domain of ACE and NEP. By doing this, the real problems can be addressed. 

In addition, ACE inhibitory peptides acquired from natural products, such as mushrooms, are not necessarily free from side effects. For instance, Lau et al. [42] stated that while the peptides had inferior ACE inhibitory activity in comparison to the commercially available anti-ACE drugs, these peptides are isolated from mushrooms, which ought to have no side effects. In order to show the peptides’ preliminary safety, a minimum of human somatic domain-specific inhibition through molecular docking analysis needs to be performed only if the peptide passed the stability test and later needs to be verified through preclinical and clinical studies. In preclinical studies, one of the tests that can be carried out is quantification of bradykinin in the rats’ plasma or serum. On the other hand, other than ACE and NEP inhibitions, the inhibition of the chymase enzyme also needs to be taken into consideration because the chymase enzyme can also convert angiotensin I to angiotensin II [14]. Furthermore, it has been shown that chronic inhibition of ACE did not alter the amount of angiotensin II in the interstitial fluid of the left ventricle of the conscious mice. Moreover, no alterations in the cardiac content of angiotensin II were detected in Lewis rats that were given the treatment for 2 weeks with either lisinopril (ACE inhibitor) or losartan (ARBs) alone or in combination. Moreover, the catalytic action of the chymase enzyme is 20-fold greater than ACE [43]. Unfortunately, commercially available ACE inhibitors are unable to inhibit the chymase pathway [44]. A combination of a dual inhibitor (C-domain ACE and NEP) and a chymase inhibitor could probably solve the patients’ problems.

## 6. Challenges Working with Naturally Derived Peptide-Based ACE Inhibitors

Although peptide-based ACE inhibitors isolated from natural products seem attractive, they need to go through many challenges before they can be implemented for clinical use. One of the biggest challenges is the instability of the peptide against gastrointestinal enzymes. Alternatively, researchers can design the peptide sequences in the presence of ACE inhibitory favourable amino acids, such as amino acids ‘W’ or ‘R’, at the C-terminal of peptides and verify their stability against the gastrointestinal enzymes. This is one of the fastest ways to create favourable ACE inhibitory peptides. To design ACE inhibitors based on the structure–activity relationship, the proline amino acid at the C-terminal of the peptide is mostly preferred. The advantage of having proline at the C-terminal is that this hydrophobic amino acid is attracted to the ACE binding sites, and the peptide is stable against enzymatic reaction because, in the presence of proline, the Keil rule is obeyed. The Keil rule is defined as trypsin (an enzyme) cleaving next to amino acid arginine (R) or lysine (K), but not before proline (P) [14]. This is an added advantage for a peptide with proline. It has been shown that most of the ACE non-domain specific inhibitors, such as captopril, lisinopril, and enalapril, have proline interacted with the ACE’s P_2_’ site [1]. The idea most likely came from the hydrophobicity of the active site. This is not surprising because the hydrophobic active site of ACE would further easily interact with hydrophobic amino acids like proline [45]. Since it has been shown that the presence of tryptophan, which is also a hydrophobic amino acid, at the C-terminal of the peptide could selectively inhibit the C-domain of ACE, scientists can create short peptide sequences with the presence of this amino acid and study the domain-specific inhibitory activity. As mentioned above, the IC_50_ of ACE inhibitory peptides isolated from natural compounds are mostly inferior to the commercially available ACE inhibitors. This is the next big challenge while working to develop peptide-based ACE inhibitors. Dipeptide VW and tripeptide GVR had IC_50_ in the micromolar range, while in human plasma, rarely, micromolar concentration is achieved where nanomolar concentration is frequently observed. For example, captopril, lisinopril, and enalapril together with RXPA380 had IC_50_ in a nanomolar range, mostly in single or double digits. Lisinopril, enalapril, captopril, and RXPA380 had IC_50_ of 2.2 nM, 6.3 nM, 14 nM, and 3 nM for C-domain inhibition, respectively [1]. The IC_50_ value at a nanomolar range is needed because at the active site of ACE, competition between the inhibitor/s and angiotensin-I takes place. Only the compound that is ‘stronger’ than angiotensin-I can bind to the active site and block the conversion of angiotensin-I to angiotensin-II. For this, the compound with a lower IC_50_ value than angiotensin-I is needed to competitively bind to the ACE’s active site. However, some peptides at a micromolar range of IC_50_, including tripeptide GVR, IPP, VPP, and many more, reduced the systolic blood pressure in spontaneously hypertensive rats (SHRs). There are other pathways other than ACE inhibition that regulate blood pressure. The other antihypertensive pathways that can be affected by peptides isolated from natural products are endothelin-converting enzyme (ECE) and endothelin 1 (ET-1) release inhibition, arginine–nitric oxide pathway inhibition, renin inhibition, and calcium channel blocking and angiotensin receptor blocking. Therefore, this might clarify the reason why several peptides with a high IC_50_ value for ACE inhibition in vitro can produce a good blood pressure lowering effect in the SHRs animal model [14]. It is also possible that the long peptide sequence, after being digested by gastrointestinal enzymes in vivo and reduced to smaller fragments of peptides, simultaneously inhibits other mechanisms and, at the same time, produces non-specific binding/s at other unrelated proteins. Other than bringing down the blood pressure, certain non-specific bindings can lead to the emergence of other side effects. Although being off-target is an issue, there are products based on peptides derived from natural products developed for human use. One of these is exenatide (Byetta™), which is isolated from the saliva of a lizard known as the Gila monster and used for type 2 diabetes mellitus. Captopril is also a peptide-based drug [46]. The latest peptide-based product development in the field of hypertension is the production of synthetic angiotensin II, which was granted an approval by the FDA in the year 2017. This synthetic peptide is used for increasing blood pressure through IV infusion in adult patients suffering from septicemia or other distributed shock [47]. In conclusion, based on this narrative review, future research should focus on comprehensive strategies in order to reduce high blood pressure in hypertensive patients without affecting the bradykinin degradation. In the end, the isolated or designed ACE inhibitory peptides or non-peptide-based compounds need to address the hypertensive patients’ real problems: chronic dry cough and angioedema.

## Data Availability

Not applicable.
